# The Alzheimer’s Disease Amyloid-Beta Hypothesis in Cardiovascular Aging and Disease: *JACC* Focus Seminar

**DOI:** 10.1016/j.jacc.2019.12.033

**Published:** 2020-03-03

**Authors:** Dimitrios A. Stakos, Kimon Stamatelopoulos, Dimitrios Bampatsias, Marco Sachse, Eleftherios Zormpas, Nikolaos I. Vlachogiannis, Simon Tual-Chalot, Konstantinos Stellos

**Affiliations:** a Cardiology Department, Democritus University of Thrace, Alexandroupolis, Greece; b Department of Clinical Therapeutics, National and Kapodistrian University of Athens School of Medicine, Athens, Greece; c Biosciences Institute, Faculty of Medical Sciences, Newcastle University, Newcastle upon Tyne, United Kingdom; d Medical School, Goethe University Frankfurt, Frankfurt am Main, Germany; e Department of Cardiology, Freeman Hospital, Newcastle Hospitals NHS Foundation Trust, Newcastle upon Tyne, United Kingdom; f NIHR Newcastle Biomedical Research Centre, Newcastle University and Newcastle upon Tyne NHS Foundation Trust, Newcastle upon Tyne, United Kingdom

**Keywords:** Alzheimer’s disease, amyloid-beta, amyloid precursor protein, atherosclerosis, cardiovascular disease, cardiovascular therapy, cerebral amyloid angiopathy, coronary artery disease, endothelial cells, leukocytes, platelets, prognosis, vascular dementia, vascular stiffness, Aβ, amyloid-beta, ACS, acute coronary syndrome, AD, Alzheimer’s disease, ApoE^−/−^, apolipoprotein E-deficient, APP, amyloid precursor protein, BACE, beta amyloid cleaving enzymes, CAA, cerebral amyloid angiopathy, CAD, coronary artery disease, CVD, cardiovascular disease

## Abstract

Aging-related cellular and molecular processes including low-grade inflammation are major players in the pathogenesis of cardiovascular disease (CVD) and Alzheimer’s disease (AD). Epidemiological studies report an independent interaction between the development of dementia and the incidence of CVD in several populations, suggesting the presence of overlapping molecular mechanisms. Accumulating experimental and clinical evidence suggests that amyloid-beta (Aβ) peptides may function as a link among aging, CVD, and AD. Aging-related vascular and cardiac deposition of Αβ induces tissue inflammation and organ dysfunction, both important components of the Alzheimer’s disease amyloid hypothesis. In this review, the authors describe the determinants of Aβ metabolism, summarize the effects of Aβ on atherothrombosis and cardiac dysfunction, discuss the clinical value of Αβ1-40 in CVD prognosis and patient risk stratification, and present the therapeutic interventions that may alter Aβ metabolism in humans.

## Highlights

• Aging is the most important risk factor for the development of cardiovascular disease and dementia. Yet, the overlapping underlying mechanisms are not completely appreciated.
• Aβ, an aging-induced peptide and the hallmark of the amyloid hypothesis of Alzheimer’s disease, constitutes an independent cardiovascular risk factor.
• We review the determinants and the role of Aβ in cardiovascular system and disease.
• We call for research gathering evidence on how to treat this newly recognized entity.

**Figure 1:**
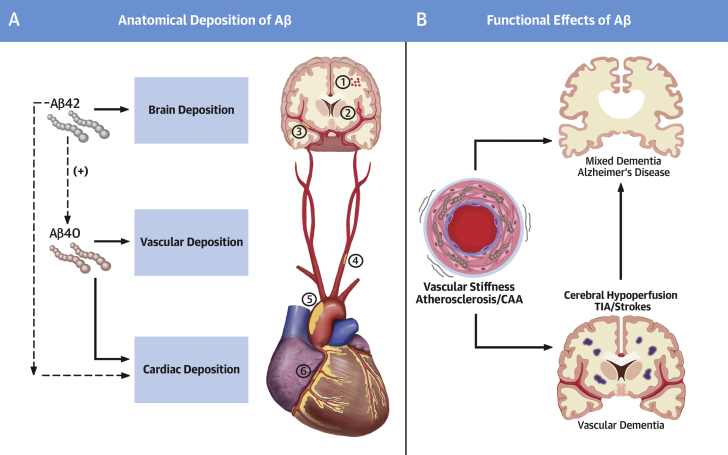
The Continuum of Cardiovascular and Neurotoxic Effects of Αβ Peptides **(A)** Amyloid-beta (Aβ) 1-42 peptides have been found in brain parenchymal and cardiac depositions and, to a lesser extent, in vessels. Depositions composed of Αβ1-40 peptides have been described mainly in the heart and vessels including several vascular beds ranging from: **(1)** leptomeningeal and cortical vessels in cerebral amyloid angiopathy (CAA); to **(2)** cerebral microvasculature; **(3)** intracerebral arteries/circle of Willis; **(4)** carotid arteries; **(5)** aorta; and **(6)** coronary/extracerebral arteries. **(B)** Brain Αβ deposits trigger a number of events involved in neuronal dysfunction clinically manifested as cognitive decline and progressive Alzheimer’s type dementia. Cardiac depositions are associated with cardiomyocyte dysfunction. Vascular Αβ deposition induces functional changes (vascular stiffening) and promotes vascular inflammation and atherosclerosis. Aging-associated Αβ-induced cardiovascular disease leads to cerebral hypoperfusion, which is a risk factor for vascular, Alzheimer’s, or mixed dementia.

Several cardiovascular risk factors have long been associated with a greater risk for future cognitive decline in nondemented individuals (1). Control of vascular risk factors effectively reduces the incidence of dementia in both healthy and cognitively impaired individuals (2). The presence of intracerebral atherosclerotic vascular disease (3) exacerbates all types of dementia and has been independently associated with worse cognitive performance even in nondemented individuals (4). These observations indicate that the aging-related inflammatory nature of both atherosclerosis and dementia involves multiple common cellular and molecular mechanisms. Recent accumulating evidence points toward the existence of a possible nonexclusive shared systems biology process that may drive aging-associated diseases, atherosclerotic cardiovascular disease (CVD), and dementia (Figure 1).

Production and accumulation of amyloid-beta (Aβ) peptides in the brain are considered the hallmark of Alzheimer’s disease (AD) amyloid hypothesis (5). The prototypic cerebrovascular disease associated with Αβ40 deposits is cerebral amyloid angiopathy (CAA) (6). CAA describes a group of aging-associated brain disorders with characteristic pathological findings of amyloid deposits predominantly in the arteriolar wall. Clinical and imaging features of CAA vary from asymptomatic microbleeds to severe hemorrhage, neurological deficits, cognitive impairment, dementia, and death. Defective perivascular drainage of neuronal-derived Aβ is probably the main mechanism of Αβ deposition. Among Aβ peptides, Αβ1-40 is the main peptide involved in the pathogenesis of CAA, whereas Αβ1-42 is mainly involved in development of AD. The vascular preference of Aβ1-40 has led to the hypothesis that this molecule may exert proinflammatory properties not only in cerebral but also in peripheral vasculature, mediating arterial disease as depicted in Figure 1, suggesting a continuum of Aβ1-40 deposits in the circulatory system ranging from leptomeningeal and cortical cerebral microvasculature (CAA) to intracerebral, carotid, aortic, or coronary vascular wall or heart. Interestingly, in contrast to studies examining associations between Aβ1-40 plasma levels and cardiovascular disease, studies assessing the association of plasma Αβ1-40 with cognitive function have not yielded consistent results (7). The detrimental properties of Αβ1-40 species on vascular brain pathology affecting memory and cognition secondarily to microvasculature damage rather than through direct neurotoxicity, may explain this discrepancy.

In this review, we present contemporary evidence that links Αβ peptides with vascular inflammation and a wide range of associated extracerebral atherosclerotic manifestations and myocardial dysfunction, as well as adverse CVD outcomes and mortality (Central Illustration). Based on this evidence, we discuss the potential clinical utility of Αβ1-40 as a biomarker for risk stratification for mortality and present therapeutic interventions that may alter Αβ accumulation.

**Central Illustration:**
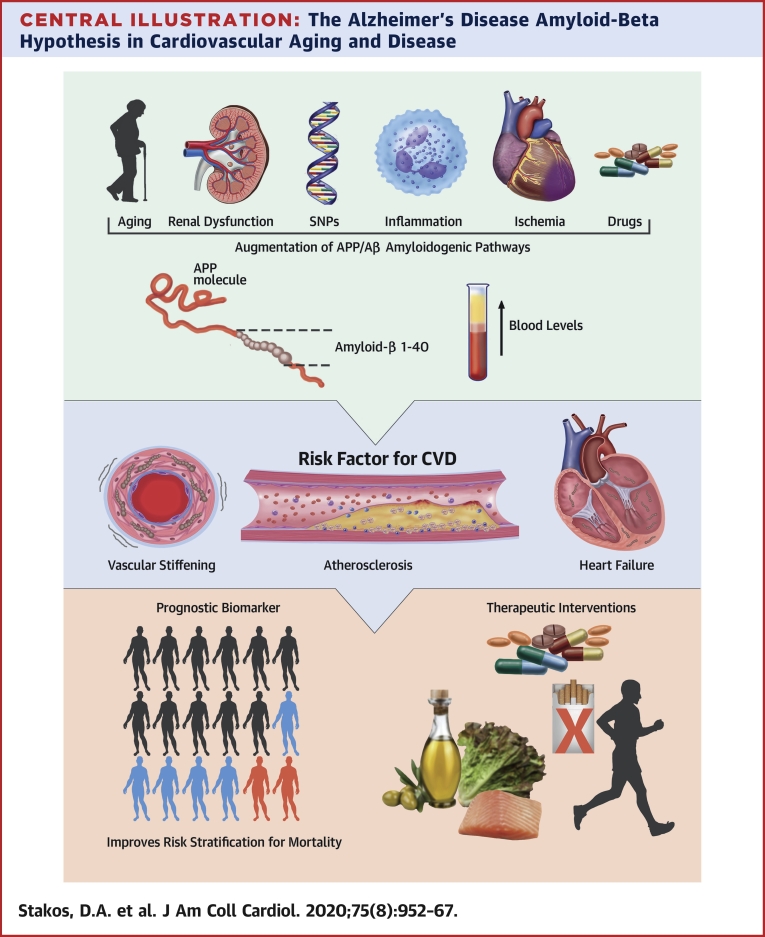
The Alzheimer’s Disease Amyloid-Beta Hypothesis in Cardiovascular Aging and Disease Several factors alter APP/Aβ metabolism by promoting amyloidogenic pathways leading to increased Αβ1-40 blood levels. Subsequent deposition of Αβ1-40 in heart and vessels induces cell damage, accelerating arterial stiffening, atherosclerosis, and cardiac dysfunction, which are manifestations of cardiovascular aging and disease. Epidemiological evidence supports the clinical relevance of these effects. Αβ1-40 blood levels fulfill several criteria as a cardiovascular prognostic biomarker for risk stratification. Lifestyle and medical interventions interfere with Αβ1-40 levels. Aβ = amyloid-beta; APP = amyloid precursor protein; CVD = cardiovascular disease; SNP = single-nucleotide polymorphism.

## Amyloid Precursor Protein and Aβ Metabolism

Aβ peptides are proteolytic fragments of amyloid precursor protein (APP), an integral membrane protein (8,9). The APP gene produces 3 major splice variants (10), APP695, APP751, and APP770, produced in neurons, endothelial cells, and platelets, respectively. The exact physiological function of this well-conserved, site-specific APP/Αβ pathway is not fully elucidated, but it is associated with natural antimicrobial defense (11) and coagulation cascade proteolytic events (12). The latter is mediated by a Kunitz-type serine protease inhibitor domain contained in APP751 and APP770 molecules.

**Figure 2:**
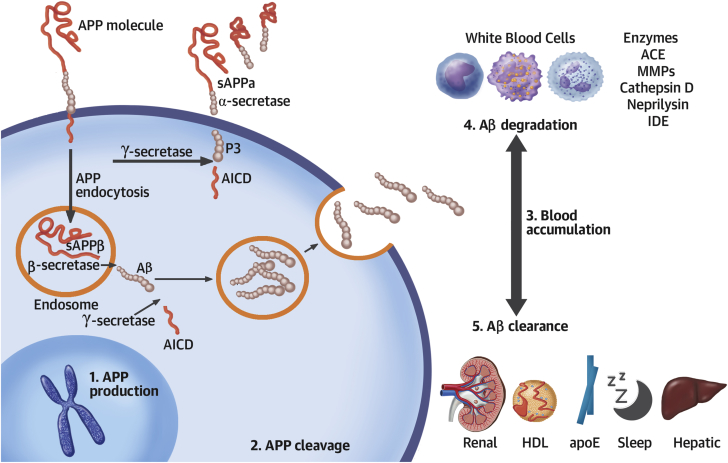
APP and Αβ Metabolism Following **(1)** amyloid precursor protein (APP) gene transcription, **(2)** APP is cleaved in the nonamyloidogenic pathway (plasma membrane) by α- and γ- secretases or in the amyloidogenic pathway (endosomes) by β- and γ- secretases. The later pathway generates amyloid beta (Αβ) peptides that are released extracellularly. **(3)** Αβ accumulation in blood or tissues may result from enhanced production/cleavage or by **(4)** impaired degradation and/or **(5)** clearance. ACE = angiotensin converting enzyme; AICD = amyloid precursor protein intracellular domain; apoE = apolipoprotein E; HDL = high-density lipoprotein; IDE = insulin degrading enzyme; sAPP = soluble amyloid precursor protein.

APP can be initially cleaved by α-secretases generating nonamyloidogenic products depending on its location on plasma membrane, the site of processing (membrane or endosomes), and environmental pH (13), or by β-secretases, also known as beta amyloid cleaving enzymes (BACE) (Figure 2). The β-secretase–mediated cleavage of APP retains the integrity of Αβ fragments within the remaining C99 peptide, while C99 subsequent cleavage by γ-secretases releases Aβ peptides (14). C99 cutting site by γ-secretases depends on the location of processing (endosomes or Golgi network) and generates amino acid peptides of length 40 (Αβ1-40 mostly found in vascular lesions) and 42 (Αβ1-42, mainly found in AD-associated brain lesions), as well as the intracellular domain of APP (Figure 2). Several factors, including aging, inflammation, renal dysfunction, ischemia, polymorphisms, and drugs, increase circulating levels and subsequent tissue deposition of Αβ by augmenting APP production and processing or by decreasing Αβ clearance and degradation (Figure 2, Online Tables 1 to 3). Under normal conditions an equilibrium exists between Aβ production and removal in various compartments inside or outside of the central nervous system (15). Deregulation of this equilibrium may lead to accumulation of Αβ1-40 in blood, vascular wall, and heart tissues, which has been associated with CVD.

## Systemic Accumulation of Aβ and CVD

### Peripheral vascular Aβ abundance

Although APP processing in different cell types gives rise preferentially to Αβ1-40 or -42 (16), it is not known what drives this differential final processing of the amyloidogenic pathway of APP. In cases of CAA, neuronal-derived Αβ (either Αβ1-40 or -42) fails to drain away from the leptomeningeal vessels, capillaries, and brain parenchyma (17). This defective depletion leads to its accumulation in brain arterioles. Αβ deposits are observed in the tunica media in close proximity as well as inside of the smooth muscle cells and in the adventitia, avoiding endothelial cells even at higher degrees of CAA (18,19). Because impairment of adventitial lymphatic capillaries in peripheral vessels also aggravates atherosclerosis, the role of lymphatic drainage in Aβ-related cardiovascular disease should be further explored. In peripheral atherosclerotic lesions, Αβ deposits consist almost exclusively from the Αβ1-40 species (20). Using mass spectrometry, Aβ1-40 peptide was on average 100 times more abundant than Aβ1-42 in human aortic atherosclerotic plaques (21). The 2-peptide-amino-acid-longer species Αβ1-42, being more hydrophobic and fibrillogenic, is the main amyloid peptide found in parenchymal lesions of AD; however, its “vascular” involvement is limited to deposits in pericapillary spaces and glia limitans, parenchymal brain vessels, and leptomeningeal vessels. Yet, overexpression of Αβ1-42 promotes Αβ1-40 vascular depositions in the brain (22), and factors that alter the Αβ1-40/-42 ratio, such as human apolipoprotein E4 (23), favor amyloid deposits in the form of CAA compared with parenchymal plaques. This differential tissue preference of Aβ species may be explained by the following observations: 1) using 3D models of cerebrovascular vessels, researchers have recently demonstrated that HDL and apolipoprotein E (ApoE) synergistically promote vascular clearance of Aβ1-42 more than that of Aβ1-40 (24); 2) Αβ1-40 is produced in significant amounts from platelets, plaque invading macrophages (25), endothelial cells (26), and vascular smooth muscle cells (27); and 3) different ApoE isoforms, which are proteins with an impact in cholesterol transport system, seem to differentially regulate Aβ production, aggregation, and clearance (28). More specifically, ApoE4 may inhibit Aβ clearance by competitively binding to the low-density lipoprotein receptor-related protein 1, and its presence has been associated with brain Αβ accumulation and increased AD risk. Interestingly, ApoE seems to affect also Αβ kinetics in blood (29).

### Aβ and subclinical vascular disease

**Figure 3:**
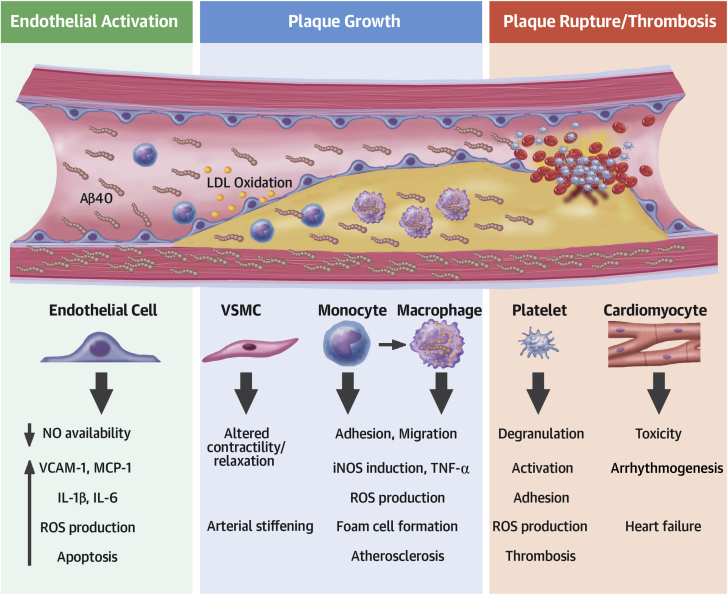
Detrimental Cellular and Molecular Effects of Aβ1-40 in the Cardiovascular System Excess in blood Αβ1-40 levels exerts detrimental effects in vascular and blood cells promoting endothelial activation, atherosclerosis, and atherothrombosis. IL = interleukin; iNOS = inducible isoform of nitric oxide synthases; LDL = low-density lipoprotein; MCP = monocyte chemoattractant protein; NO = nitric oxide; ROS = reactive oxygen species; TNF = tumor necrosis factor; VCAM = vascular cell adhesion molecule; VSMC = vascular smooth muscle cells.

Aβ1-40 is critically involved in vascular aging. SIRT1, a class III histone deacetylase, plays a pivotal protective role in vascular aging (30) as it up-regulates α-secretase activity shifting Aβ metabolism towards the non-amyloidogenic pathway (Figure 2). However, activation of the amyloidogenic pathway results in impairment of the vasodilating properties of small arterioles by enhancement of endothelin-1 expression (31), reduction of eNOS activity and endothelium-dependent vasodilation, enhancement of oxidative stress (32), and increased responsiveness to vasoconstrictors (33) (Table 1, Figure 3). Further, Aβ oligomers may inhibit telomerase activity leading to telomere shortening (34), which actively promotes vascular aging. This experimental evidence generates the hypothesis that increased Aβ systemic concentrations may be associated with measurable, accelerated arterial aging and deteriorated vascular function and structure in humans. Arterial pulse wave velocity is a well-established, noninvasive marker of arterial stiffness and vascular aging (35). Interestingly, the severity of cerebral β-amyloid deposition measured by positron emission tomography scan and its change over 2-year follow-up was associated with higher pulse wave velocity in nondemented elderly adults (36,37). To assess whether Aβ1-40 is involved in early processes of arterial disease and aging, we prospectively examined changes in pulse wave velocity and plasma Aβ1-40 in 107 young to middle-aged healthy adults (mean age 46.2 years), clinically followed for 5 years (38). We found that the 5-year change of plasma Aβ1-40 levels was an independent determinant of the 5-year change in aortic stiffness. Because Aβ1-40 deposits have been found in carotid human atherosclerotic plaques (25,39) and aortas (21), we examined whether plasma Aβ1-40 levels are associated with subclinical atherosclerosis in a population of 394 individuals with a wide range of CVD risk profiles. After adjustment for age, traditional CVD risk factors, and renal function, increased Αβ1-40 was independently associated with higher carotid intima-media thickness, lower ankle-brachial index, and the severity and extent of arterial damage assessed in the carotid and femoral arteries, aorta, and coronary circulation (38). Plasma Aβ1-40 was also associated with the severity of coronary artery calcium score in a sample of 3,266 adults from the Dallas Heart Study without clinically overt CVD (40).

**Table 1:** Role of APP and Aβ in Cardiovascular Biology and Disease

Molecule	Study Design	Tissue or Cell-Specific Effects	Ref. #
**Endothelial Cells**			

APP	Murine and human cell line	Increased protein levels of proinflammatory mediators (COX-2, VCAM-1) and increased secretion of IL-1β and Aβ1-40 through Src kinase signaling pathway	(69)
Aβ1–40	Human cell line	Increased expression of inflammatory genes (MCP-1, GRO, ΙL-1β, and IL-6) through JNK-AP1 signaling pathway	(48,70)
Aβ1–40	Rat cell line	Increase of endoplasmic reticulum stress through unfolded protein response	(71)
Aβ1–40	Human, mouse, rat, and bovine cell line	Inhibition of the KCa^2+^ channel opening and reduced Ca^2+^ efflux	(71,72)
Aβ1–40	Human and rat cell line	Activation of caspase-dependent and -independent apoptosis through caspase 12 and cytochrome c	(48,71)
Aβ1–40 Aβ1–42 Aβ25–35	Human, mouse, bovine, and porcine cell line, rat arteries	Inhibition of NO signaling in a concentration-manner through interaction with CD36	(72,73)
Aβ1–40 Aβ1–42	Human cell line	Signature transcriptomic of essential endothelial function affected	(48)

**Smooth Muscle Cells**			

Aβ1–42	Human and porcine cell line	Decrease in sGC activity and cGMP production	(73)

**Cardiomyocytes**			

Aβ1–40 Aβ1–42	Murine and human cell line	Decrease of cell viability	(48)

**Monocytes**			

APP	Murine and human cell line	Recruitment of tyrosine kinases Lyn and Syk to APP during β1 integrin-mediated adhesion of monocyte through tyrosine kinase mechanism	(69,74,75)
Aβ1–42	Human monocytes	Differentiation of monocytes into macrophages	(76)
Aβ1–40 Aβ1–42 Aβ25–35	Human monocytes Human cell line	Hypersecretion of inflammatory cytokines (TNF-α and IL-1β) and chemokines (MCP-1, IL-8, MIP-1 α, and CCR5) through activation of ERK-1/-2	(43,76, 77, 78, 79)
Aβ1–40 Aβ1–42 Aβ25–35	Human and murine cell line	Secretion of ROS	(79)
Aβ1-40	Human cell line	Migration of monocyte through ERK-1/-2 and RAGE receptor	(74,80)
Aβ1-40 Aβ1-42	Human cell	Opsonization of lipoproteins enhances their uptake by human monocytes, resulting in cholesterol accumulation	(81)

**Macrophages**			

Aβ1–40	Murine cell line	Enhanced nitrite production in the presence of IFN-γ macrophage activation	(25)
Aβ1-40 Aβ1-42	Human cell	Opsonization of lipoproteins enhances their uptake by macrophages, resulting in cholesterol accumulation Accelerated formation of foam cells	(81)
Aβ1–42	Macrophages from CD36^−/−^ mice	Production of ROS and proinflammatory cytokines IL-1β and TNF-α through CD36 signaling	(82,83)

**Platelets**			

sAPP695α sAPP751α sAPP770α	Human platelet	Inhibition of platelet aggregation and secretion	(84)
Aβ1–40	Amyloid properties induced in unrelated proteins to stimulate human and murine platelets	Platelet aggregation through either a CD36-p38^MAPK^-TXA_2_ or a glycoprotein Ibα pathway	(85)
Aβ1–40 Aβ25–35	Human platelet	Platelet aggregation with Ca^2+^ mobilization and PLC γ 2-PKC pathway activation	(86)
Aβ25–35	Human and murine platelet	Platelet activation through RhoA-dependent modulation of actomyosin Increase in intracellular Ca^2+^, leading to dense granule release and ADP secretion	(87,88)
Aβ1–40 Aβ1–42 Aβ25–35	Human and murine platelet	Platelet adhesion and spreading through the elongation of filopodia and lamellipodia	(89,90)
Aβ1-42	Human plasma	Thrombin generation in an FXII-dependent FXI activation	(91)
Aβ1–40	Human and murine platelet	ROS generation and cell shrinkage	(89)
APP	Overexpression of human APP isoform 770 in mice platelets	Marked inhibition of thrombosis in vivo	(85)
APP	Overexpression of human APP isoform 751 in mice	Prothrombotic phenotype in vivo	(61)

APP = amyloid precursor protein; Aβ = amyloid beta; CCR5 = chemokine receptor type 5; cGMP = cyclic guanosine monophosphate; COX = cyclooxygenase; ERK = extracellular signal–regulated kinase; FX = coagulation factor.; GRO = growth-related oncogene; IL = interleukin; IFN = interferon; JNK-AP = c-Jun N-terminal kinase–activator protein; MCP = monocyte chemo-attractant protein; MIP = macrophage inflammatory protein; NO = nitric oxide; PKC = protein kinase C; PLC = phospholipase C; RAGE = receptor advanced glycation end products; ROS = reactive oxygen species; sGC = soluble guanylyl cyclase; TNF = tumor necrosis factor; TXA2 = thromboxane A2; VCAM = vascular cell adhesion molecule.

Overall, these findings are indicative of direct and indirect roles of Aβ1-40 in accelerated arterial aging, atherosclerosis at various stages, and vascular beds, taking place long before the establishment of clinically overt CVD.

### Αβ1-40 in coronary artery disease

Circulating Aβ1-40 levels were independently associated with the presence of angiographically documented stable coronary artery disease (CAD) in 2 independent cohorts consisting of 514 and 396 patients (38). This association was confirmed in subsequent studies, including adults with normal cognitive function or patients with AD (41,42).

Experimental evidence indicates that Aβ peptides may be actively involved in downstream pathways leading to plaque rupture, thrombosis, and subsequent clinical manifestations of the acute coronary syndrome (ACS) (Figure 3). Αβ1-40 stimulates platelet activation and adhesion in humans and mice (Table 1) and induces release of matrix metalloproteinases by human monocytes to increase plaque vulnerability (43). Interestingly, in a myocardial infarction rat model, early surges in plasma sAPP770 concentrations preceded the release of cardiac injury enzymes (26), while plasma sAPP was also increased in patients with ACS (26), suggesting that enhanced APP/Aβ processing and subsequent release of sAPP770 and Αβ1-40 may trigger plaque rupture or its sequalae in ACS. In support of this hypothesis (Figure 3), we recently reported that in 2 independent cohorts of patients with non-ST-segment elevation ACS, higher blood Aβ1-40 levels were associated with worse risk profile, including a higher GRACE (Global Registry of Acute Coronary Events) score high sensitivity cardiac troponin T and lower systolic blood pressure and estimated glomerular filtration rate (44), implying a concentration-dependent relation of Aβ with the severity of ACS. Overall, the results of these studies provide conceptual proof that Aβ metabolism is enhanced in CAD and Aβ1-40 levels in blood are increased and associated with its clinical presentation.

## Aβ1-40, Mortality, and Risk Stratification

### General population

High plasma Aβ1-40 concentrations were independently associated with increased risk of mortality in 1,254 elderly subjects after adjustment for CVD risk factors and frailty (45). However, significance was lost after adjustment for cystatin C, suggesting that this association may be mediated by differences in renal function and/or inflammatory status. The prognostic value of circulating Aβ1-40 in nonelderly subjects from the general population as well as its reclassification potential remain unknown.

### Coronary artery disease

We have recently shown that circulating Aβ1-40 blood levels measured in 2 independent populations of patients with stable CAD were predictive of a 3-fold increased risk of cardiovascular death for highest versus lowest quartile (38). Importantly, adding Aβ1-40 improved risk stratification over the best predictive model by reclassifying 22% of the population to correct risk categories for cardiovascular mortality.

In-hospital and midterm mortality in patients with ACS vary considerably from <1% to >8% according to risk score calculators (46,47). However, no indexes of vascular inflammation are currently included in risk estimation scores such as the widely recommended GRACE score assessing mortality (46,47). To this end, we have demonstrated that measuring Aβ1-40 in patients with non–ST-segment elevation ACS improves prognostic assessment and provides incremental reclassification value over the GRACE score (44). A single measurement of circulating Aβ1-40 at presentation was independently associated with mortality in both cohorts (44). Importantly, Αβ1-40 substantially improved risk stratification of patients with non–ST-segment elevation ACS into correct risk categories over the GRACE score (net reclassification index 33.4% to 47.1%).

Collectively, these findings suggest that Αβ1-40 may be a clinically useful risk biomarker in stable CAD and particularly in non–ST-segment elevation ACS where Aβ1-40’s performance was complementary to that of the GRACE score, a commonly used risk score in clinical practice. However, clinical application of this peptide as a biomarker needs further research to set reference values and thus allow its investigation as part of novel prognostic algorithms in CAD.

### Αβ1-40 and cardiac function

A deregulation of the BACE1/Αβ1-40 axis was identified in the hearts of nondemented individuals with ischemic heart failure (48), whereas histology confirmed Aβ1-40 and -42 aggregates in the heart of patients with AD (49), suggesting a novel form of aging-related cardiac amyloidosis that merits further investigation. Mechanistically, both peptides exert toxic effects on cardiomyocytes resulting in poorer cell viability and apoptosis (48,49). Treatment of zebrafish embryos with Aβ1-40 peptides induces impaired vascular development and angiogenesis (50), possibly by interfering with VEGF pathway (51). Because ischemia promotes both APP up-regulation and cleavage (52), and Aβ1-40 may induce vasoconstriction and reduced endothelium-dependent vasodilatation (53), the pathogenic consequences of short- or long-term myocardial ischemia on heart failure via enhanced cardiac amyloidogenesis should be explored.

Many aspects of Aβ-related cardiac amyloidosis are supported by clinical findings. Plasma Αβ1-40 has been associated with markers of cardiac dysfunction in several clinical conditions with variable degrees of myocardial functional impairment. We have recently demonstrated that in 3,266 individuals without clinically overt CVD from the Dallas Heart Study who underwent cardiac magnetic resonance imaging, plasma Αβ1-40 was associated with increased circulating N-terminal pro–B-type natriuretic peptide and high sensitivity cardiac troponin T, indicative of involvement of this peptide in early subclinical myocardial stretch and injury (40). Interestingly, we also found an association of Aβ1-40 with lower left atrial emptying fraction after adjustment for CVD risk factors. In contrast, although stroke volume index was lower at higher levels of Aβ1-40 by univariate analysis, we observed no independent associations with more advanced cardiac abnormalities such as left ventricular systolic dysfunction or remodeling, possibly because the population under study was free of established heart disease and such late changes were not discernible. Indeed, increased plasma Αβ1-40 was found in patients with established CAD and lower left ventricular ejection fraction (38). Given that Aβ1-40 is associated with lower cardiorespiratory fitness (VO_2_ max) independently of daily activity (40) and with left atrial dysfunction, further studies are needed to assess whether lower VO_2_ max is of cardiac origin possibly related to Αβ1-40–mediated subclinical myocardial damage. Accordingly, the presence of Aβ1-40 in the heart has been associated with echocardiographic findings of early diastolic dysfunction (49). Furthermore, in a prospective study of 939 patients with heart failure showing reduced or preserved ejection fraction, plasma Αβ1-40 levels were associated with symptoms of heart failure as described in New York Heart Association’s functional classification system (54). Because diastolic dysfunction and heart failure with preserved ejection fraction are considered prominent manifestations of myocardial aging (55), blood concentrations of Αβ1-40 may reflect the extent of its vascular and myocardial involvement in CVD. The clinical relevance of this concept is supported by recent findings showing that circulating Aβ1-40 predicts adverse clinical outcomes and mortality and improves risk stratification in patients with heart failure (54).

### Experimental evidence of the link between Aβ and CVD

A dementia-CVD continuum hypothesis is further demonstrated through the vascular involvement of dementia-prone transgenic APP mice. The Tg2576 mouse model expresses 5 times the levels of endogenous murine APP (56) and shows progressive impairment of cognitive function together with Αβ1-40–dependent (57) and ROS-mediated (53,58) endothelial dysfunction, impaired vascular reactivity, and 30% attenuation in cerebral blood flow (59). B6Tg2576 mice develop more extensive aortic lesions than control mice when fed the same atherogenic or normal diet under similar lipid profiles (60). APP23 mice, which overexpress APP and Αβ1-40, show enhanced platelet integrin activation and degranulation as well as accelerated thrombus formation (61). Dementia-prone APP23 mice crossed with atherosclerosis-prone apolipoprotein E–deficient (ApoE^−/−^) mice develop larger and more inflammatory aortic atherosclerotic lesions compared with ApoE^−/−^ mice (62). Conversely, ApoE^−/−^ mice crossed with animals lacking APP (APP^−/−^) have significantly reduced atherosclerotic plaque size in thoracic and abdominal aorta (90% and 75% reduction, respectively) compared with ApoE^−/−^ mice despite comparable cholesterol levels (63). More importantly, atherosclerotic plaques in APP^−/−^/ApoE^−/−^ mice have reduced macrophage content, increased amount of collagen, and a thicker fibrous cap indicating a more stable plaque morphology. Mechanistically, a series of experimental studies summarized in Table 1 present Αβ as a potent proinflammatory, proapoptotic, and proatherogenic molecule affecting the function of endothelial cells, platelets, vascular smooth muscle cells, and macrophages (Figure 3).

## Interventions Affecting Aβ Metabolism

### Lifestyle modifications

A healthy lifestyle, including adherence to Mediterranean diet, omega-3 fatty acids, and caloric restriction may reduce Aβ brain deposits and exert antiamyloidogenic properties (Online Table 4). We recently demonstrated that increased daily activity assessed by accelerometer recordings and lower physical fitness, as assessed by VO_2_ max, in 3,266 participants without CVD from the Dallas Heart Study were independently associated with plasma levels of Aβ1-40 (40). However, changes of Aβ peptide blood levels over time in response to physical activity have not been assessed. Yet, accumulating evidence suggests that an unhealthy lifestyle such as a high-fat diet and cigarette smoking (64) may enhance the amyloidogenic pathway (Online Table 4). These findings suggest that cardiovascular effects of lifestyle modifications may be partly mediated by altering Aβ metabolism, but further research should explore these effects in humans, particularly with regards to Aβ1-40 as a direct effector molecule in cardiovascular disease.

### Cardiovascular medical treatment

#### Statins

Experimental evidence indicates that statins reduce brain and intracellular Aβ levels in vitro and in vivo, by down-regulating its upstream pathway, reducing cellular uptake of Aβ peptides, and enhancing its clearance through the blood brain barrier (Table 2). However, results of 2 randomized clinical studies evaluating blood Αβ1-40 peptides after statin treatment were inconsistent, possibly due to statins’ effect on equilibrium between brain and circulating Αβ (Table 2).

**Table 2:** Off-Target Effects of Statins on Aβ Metabolism and Accumulation

Intervention/Condition	Cell Type/Population	Effects on Aβ Metabolism	Ref. #
Lovastatin (escalating doses 10–60 mg OD)	Double-blind, randomized, placebo-controlled clinical study of 94 patients with hypercholesterolemia, 12 weeks	Serum levels of total Aβ are reduced in a dose-dependent manner	(92)
Simvastatin (20 mg OD)	Prospective interventional clinical trial of 19 patients with AD, 12 weeks	CSF levels of alpha and beta-secretase-cleaved APP decreased, no change in plasma levels of Aβ1-42	(93)
Pravastatin (10 mg OD)	Prospective observational clinical study of 46 patients with hyperlipidemia, 6 months	No change in plasma levels of Aβ1-40 and Αβ1-42	(94)
Simvastatin (20–80 mg OD) or Atorvastatin (20–80 mg OD)	Prospective interventional randomized clinical trial of 39 patients with hypercholesterolemia, 9 months	No change in plasma levels of Aβ1-40, Aβ1-42, or total Aβ	(95)
Simvastatin (escalating 40–80 mg OD)	Prospective open-label trial of 12 patients with AD or mild cognitive impairment and hypercholesterolemia, 12 weeks	No change in plasma levels of Aβ1-40	(96)
Simvastatin Lovastatin	Neuronal cell culture, Guinea pigs	Decreased production of Aβ1-40 and Αβ1-42 in neurons *in vitro* Decreased CSF levels of Aβ1-40 (−47%) and Aβ1-42 (−62%)	(97)
Lovastatin Simvastatin	HEK cells	Inhibited dimerization of β-secretase Decreased intracellular production of total Aβ	(98)
Fluvastatin	C57BL/6 mice neurons HBME cells	Increased APP-CTF clearance to the lysosome in neurons Increased LRP-1 and Aβ uptake in HBME	(99)
Simvastatin	PBCE cells 3x Tg AD mice	Increased LRP1 and apoJ expression Reduced Aβ uptake by PBCEC Decreased production of APP-CTFs in brain capillary endothelial cells of mice neurons	(100)

Aβ = amyloid beta; AD = Alzheimer’s disease; apoJ = apolipoprotein J; APP = amyloid precursor protein; APP-CTF = amyloid precursor protein C-terminal fragment; CSF = cerebrospinal fluid; HBME = human brain micro-endothelial cells; HEK cells = human embryonic kidney cells; LRP = low density lipoprotein receptor-related protein; OD = oral dose; PBCE = porcine brain capillary endothelial cells; 3x Tg AD mice = transgenic Alzheimer’s disease mice.

#### Antihypertensive and heart failure drug treatment

Most classes of antihypertensive drugs used in clinical practice influence APP/Aβ metabolism (Table 3). Inhibition of the angiotensin-converting enzyme increases Aβ1-40 or Aβ1-42 availability due to attenuation of its breakdown (65) or through blockade of Aβ1-42 conversion to Aβ1-40 (65), respectively. Consequently, plasma levels of Aβ1-42 were found to increase after angiotensin-converting enzyme inhibition, but results of Aβ1-40 levels were not consistent, showing either increase or no change (Table 3). The favorable effects of angiotensin receptor antagonists on Aβ metabolism shown in the central nervous system (Table 3) have not been investigated on the cardiovascular system in humans, similar to the effect of β-blockers, calcium-channel blockers, and diuretic agents (Table 3).

**Table 3:** Off-Target Effects of Antihypertensives and Heart Failure Treatment on Aβ Metabolism and Accumulation

Intervention/Condition	Cell Type/Population	Effects on Aβ Metabolism	Ref. #
**ACE Inhibitors**			

Captopril	CHO cells, HEK293 cells	ACE degrades Aβ1-40 and -42 ACE inhibition increases total Aβ levels	(65)
Captopril	Tg2576 mice, Post-mortem human brain tissue	ACE converts Aβ1-42 to Aβ1-40 ACE inhibition increases Aβ1-42 deposition in human and mice neurons	(101)
Trandolapril	Tg2576 mice	Decreased brain Aβ1-40 and Aβ1-42 Increased plasma Aβ1-40 and Aβ1-42 (x2.5)	(102)
Lisinopril (2.5–80 mg daily) Enalapril (10 mg daily) Benazepril (10 mg daily)	Observational clinical study of 22 patients with mild cognitive impairment	Increased Aβ1-42 levels and Aβ1-42/-40 ratio in plasma	(103)

**ARBs**			

Losartan	SHRSP rats	Decreased content of Aβ1-40 (−30%) and Aβ1-42 (−25%) by enhancing insulin-degrading enzyme, neprilysin, and transthyretin expression in brain	(104)
Olmesartan	APP23 transgenic mice	Olmesartan prevents Aβ1-40 induced elevation of ROS Aβ burden not reduced in brain microvessels	(105)
Candesartan	Primary neuron cultures from Tg2576 mouse embryos	Prevents Αβ1-40 and -42 aggregation and Aβ1-42 oligomerization in neurons	(106)
Losartan	Tg2576 mice	Reduced plasma and brain Aβ1-42 (−20%), while no changes in Aβ1-40 levels	(102)
Candesartan, irbesartan, olmesartan, valsartan, losartan, telmisartan eprosartan	Healthy elderly Cross-sectional study (n = 871) Prospective study (n = 124)	Increased clearance of Aβ1-42 from the brain into CSF	(107)

**ARNIs**			

Sacubitril/valsartan (400 mg OD)	Double-blind, randomized, placebo-controlled clinical study of 43 healthy subjects	Treatment increased CSF Aβ1-38 peptide and plasma Aβ1-40 levels (+50%)	(108)

**B-Blockers**			

ICI 118,551 (beta-blocker used in experimental conditions)	C57 mice	β2 adrenergic receptor blockade attenuates acute stress-induced Aβ1-40 (−20%) and Aβ1-42 (−5%) in neurons	(109)
Propranolol	SAMP8 mice	Propranolol attenuates increases in Aβ1-42 and BACE1 and decreases in IDE expression by shifting APP cleavage to nonamyloidogenic pathway in neurons	(110)
Propranolol Carvedilol	Tg2576 mice	Propranolol reduces plasma and brain Aβ1-40 (−40%) and Αβ1-42 (−50%) Carvedilol reduces brain Aβ1-40 and -42 levels	(102)
Carvedilol	N2a cells	Protective against endogenous Aβ-induced neurotoxicity in neuronal N2a cells	(111)

**CCBs**			

Nilvadipine, nitrendipine, amlodipine	TgPS1/APPsw mice or B6/SJL F1 mice	Nilvadipine and nitrendipine but not amlodipine (acute treatment) reduce brain content of Aβ probably by stimulating clearance through BBB	(112)
		Nilvadipine (chronic treatment) reduces amyloid plaque burden in mouse brain	(112)
Nilvadipine, amlodipine, nifedipine, nitrendipine	TgPS1/APPsw mice	Nilvadipine and nitrendipine increase Aβ1-40 and Aβ1-42 plasma levels, while amlodipine and nifedipine had no effect on Aβ1-40 or Aβ1-42 plasma levels	(112)
Amlodipine, diltiazem, felodipine, isradipine, nifedipine, nicardipine, nimodipine, nisoldipine	H4 neuroglioma cells	Nifedipine reduces production of Aβ1-42 (−40%), by increasing α-secretase and diminishing γ-secretase activity	(113)
Nicardipine	Tg2576 mice	Nicardipine reduces plasma Aβ1-40 (−30%) and Αβ1-42 (−50%)	(102)
Nitrendipine	Primary neuron cultures generated from Tg2576 mouse embryos	Nitrendipine prevents Αβ1-40 and -42 aggregation and Aβ1-42 oligomerization in vitro	(106)

**Diuretic Agents**			

Furosemide	Tg2576 mice	Aβ1-40 and -42 brain content decreased Plasma Aβ1-40 and -42 increased (×2)	(102)
Furosemide	Neurons of Tg2576 mice	Furosemide prevents Αβ oligomerization in vitro and reduces amyloid burden (−30%) by dissociating pre-aggregated Aβ1-42 oligomers	(106)

**Hemodialysis**			

Hemodialysis	Cross-sectional study of 30 CKD patients under hemodialysis	Hemodialysis removes blood Aβ1-40 and -42 while plasma Aβ remains decreased longitudinally	(114)
Hemodialysis	Prospective study of 26 CKD patients under hemodialysis	Plasma levels Aβ1-40 (−35%) and Αβ1-42 (−22%) reduced after 1 hemodialysis session	(115)
Hemodialysis	Prospective clinical study of 30 CKD hemodialysis patients	Long-term hemodialysis leads to reduced or unchanged plasma Aβ1-40 while plasma Aβ1-42 remains unchanged or increases	(116)
Hemodialysis	Cross-sectional study of 47 patients with CKD	Plasma levels of Aβ1-40 and -42 are reduced	(117)
Peritoneal dialysis	Cross-sectional study of 30 patients with CKD	Peritoneal dialysis decreases plasma levels Aβ1-40 and -42	(118)

Aβ = amyloid beta; ACE = angiotensin-converting enzyme; ARBs = angiotensin receptor blockers; ARNIs = angiotensin receptor/neprilysin inhibitors; BBB = blood brain barrier; CCBs = calcium-channel blockers; CHO cells = Chinese hamster ovary cells; CKD = chronic kidney disease; CSF = cerebrospinal fluid; HEK cells = human embryonic kidney cells; IDE = insulin degrading enzyme; ROS = reactive oxygen species; SAMP8 = senescence-accelerated mouse model; SHRSP rats = stroke-prone spontaneously hypertensive rats.

A new heart failure drug class, the angiotensin receptor-neprilysin inhibitors, involves the inhibition of neprilysin, which is an Αβ degrading enzyme and thus may increase Aβ1-40 plasma levels (Table 3). In light of new evidence showing that Aβ1-40 blood levels are associated with increased mortality in patients with heart failure not receiving angiotensin receptor-neprilysin inhibitors (54) and that Aβ1-40 is widely expressed in the myocardium of patients with heart failure (48), it remains unknown whether some beneficiary effects of angiotensin receptor-neprilysin inhibitors may be partly offset due to increased systemic Aβ1-40 availability. This may be particularly important in regard to long-term outcomes, as deposition diseases need time to evolve. Peritoneal dialysis and hemodialysis reduce plasma levels of Aβ1-40 and -42 (Table 3), supporting the significance of Αβ renal clearance indicating a definite interventional target on Aβ1-40 availability.

#### Antithrombotic agents

Although some evidence indicates that at low concentrations, anticoagulant agents may increase Aβ metabolism, most experimental studies indicate that mainly due to their glycosaminoglycan structure, heparin and enoxaparin inhibit Aβ neurotoxic effects by affecting APP function and BACE1 activity (Online Table 5). However, whether these protective effects are extended systemically to the cardiovascular system merits further investigation. In contrast, 1 experimental study showed that treatment of C57BL/6 mice with anticoagulants greatly increased plasma levels of Aβ (>20-fold) (66) through down-regulation of the factor XII–factor VII pathway, which is involved in Aβ degradation (66). Clopidogrel or aspirin may interfere with APP/Aβ generation from platelets, but further studies are needed to confirm this relationship (Online Table 5).

Finally, although most phase III trials assessing antiamyloid-specific, targeted therapies were negative regarding efficacy in AD (67), their impact on CVD is unknown and merits further investigation.

## Conclusions and Future Directions

Several issues merit clarification. Although patients with CAD are more likely to develop AD-like neuropathological lesions than those without CAD (68), whether atherogenesis occurs in parallel or independently from brain parenchyma amyloid load in humans is unknown. In B6Tg2576 mice, brain Aβ load is positively correlated with the area of aortic atherosclerotic lesions, while APP23/ApoE^−/−^ mice developed aortic atherosclerotic lesions well before any parenchymal brain depositions (62). The association between Αβ1-40 and normal or premature cardiovascular aging needs to be further elucidated. Understanding the mechanisms responsible for the vascular preference of Aβ1-40 over -42 can elucidate the precise biological role of this peptide in the complex pathophysiology of vascular inflammation.

A pathophysiological role of Aβ1-40 across the continuum of cardiovascular disease is suggested through its independent association with a broad spectrum of vascular and cardiac involvement from early functional vascular alterations and subclinical atherosclerosis to overt symptomatic CAD, ACS, and heart failure. This is robustly supported by experimental evidence that APP and Aβ1-40 are critically involved in vascular inflammation, vascular and cardiac aging, and atherothrombosis. The association of Aβ1-40 with mortality has been consistently shown in a total population of about 5,000 patients in 6 independent cohorts derived from 8 countries. Thus, Aβ1-40 fulfills several criteria for consideration as a new biomarker for risk stratification in cardiovascular disease, including proof of concept, clinical utility, prospective validation, incremental and reclassification value for risk prediction, and ease of use. The implementation of a universally accepted method of sampling, preparation, storage, and measurement of circulating Aβ1-40 in plasma and the definition of normal and reference values as well as the conduction of studies with strict protocols of measurement in well-defined populations will allow the clinical application of this peptide as a new risk biomarker in patients with established cardiovascular disease. Interestingly, the association of Aβ1-40 with subclinical functional vascular alterations in healthy individuals and its association with all-cause mortality in the general population indicate that it should be further tested as a possible biomarker of cardiovascular risk in primary prevention as well. Most importantly, multiple lines of evidence clearly indicate that manipulating APP/Aβ turnover and aggregation or blocking its inflammatory reactions is feasible, potentially improving our understanding and means to simultaneously protect the brain, heart, and vessels during physiological or premature aging.
