# Supplementary material for: The Alzheimer’s Disease Amyloid-Beta Hypothesis in Cardiovascular Aging and Disease: JACC Focus Seminar
Source: J Am Coll Cardiol. 2020 Mar 3;75(8):952–67. doi: 10.1016/j.jacc.2019.12.033 (PMC7042886; doi:10.1016/j.jacc.2019.12.033)
Supplement: Online Data [file mmc1.pdf]

Online appendix

The Alzheimer's Disease Amyloid-Beta Hypothesis in Cardiovascular Aging and Disease

JACC Focus Seminar

Dimitrios A. Stakos[a,*], MD; Kimon Stamatelopoulos[b,c*], MD; Dimitrios Bampatsias[b], MD cand.; Marco Sachse[c,d], MD cand.; Eleftherios Zormpas[c], MSc; Nikolaos I. Vlachogiannis[c], MD; Simon Tual-Chalot[c], PhD; and Konstantinos Stellos[c,e,f], MD.

[a]Cardiology Department, Democritus University of Thrace, Alexandroupolis, Greece
[b]Department of Clinical Therapeutics, National and Kapodistrian University of Athens School of Medicine, Athens, Greece
[c]Biosciences Institute, Faculty of Medical Sciences, Newcastle University, Newcastle Upon Tyne, UK
[d]Medical School, Goethe University Frankfurt, Frankfurt am Main, Germany
[e]Department of Cardiology, Freeman Hospital, Newcastle Hospitals NHS Foundation Trust, Newcastle Upon Tyne, UK
[f]NIHR Newcastle Biomedical Research Centre, Newcastle University and Newcastle upon Tyne NHS Foundation Trust, Newcastle upon Tyne, UK.
* these authors contributed equally

Short title: Aβ in CVD

Correspondence:
Konstantinos Stellos, MD; Biosciences Institute, Faculty of Medical Sciences, Newcastle University, International Centre for Life, Central Parkway, Newcastle Upon Tyne NE1 3BZ, United Kingdom. Phone: +44(0) 191 241 8629. Fax: 44 (0)191 241 8666. Email: konstantinos.stellos@ncl.ac.uk

Methods

For the purpose of this review we performed a thorough research in MEDLINE database from October 2018 to July 2019. We used various combinations of keywords that included ([Amyloid beta] OR [Abeta] OR [Aβ] OR [APP]) AND ([cardiovascular disease] OR [atherosclerosis] OR [heart failure] OR [cardiovascular risk factor] OR [clearance] OR [metabolism] OR [endothelial cells] OR [endothelium] OR [vascular] OR [heart] OR [smooth muscle cells] OR [platelets] OR [macrophages] OR [foam cells] OR [inflammation] OR [leukocytes] OR [cardiomyocytes] OR [LDL] OR [oxidized LDL] OR [smoking] OR [physical activity] OR [statins] OR [lipid lowering] OR [antihypertensive treatment] OR [ACE inhibitors] OR [ARBs] OR [b-blockers] OR [ARNIs] OR [diuretics] OR [CCBs] OR [hemodialysis] OR [anticoagulants] OR [antiplatelet] OR [dementia] OR [Alzheimer’s disease] OR [cerebral amyloid angiopathy] OR [CAA]).

Supplemental Table 1. Determinants of amyloidogenic APP metabolism			
**APP production**			
**Effector**	**Effect**	**Tissue / cell specificity**	**Source**
**Aging**	↑	Human brain	(1)
**IL-1 β**	↑	Human umbilical vein endothelial cells, rat neuronal and glial cells	(2,3)
**IL-6/IL-6R**	↑	Human neuroblastoma cells	(4)
**Cholesterol**	↑	Murine neuroblastoma cells	(5)
**LPS**	↑	Murine microglial cells	(6)
**Sphingolipids**	↑	CHO cells	(7)
**ApoE4 > ApoE3 > ApoE2**	↑	Human neuronal cells co-cultured with murine embryonic fibroblast	(8)
**Focal ischemia**	↑	Rat brains tissues	(9)
**APP processing**			
**Aging**	Increases BACE1 activity	Murine and monkey brains and human cerebellum, temporal and frontal cortex	(10)
**IFN-γ**	Increases BACE1 expression	Human astrocytoma cells and murine primary astrocytes	(11)
**TNF-α**	Increases BACE1 expression	Murine primary astrocytes	(12)
**ARF6**	Promotes APP cleavage by sorting BACE1 to early endosomes	Murine embryonic fibroblast	(13)
**Caloric restriction**	Increases SIRT1expression leading to increased α-secretase activity	Murine neurons and hamster ovarian cells	(14)
**LPS**	Increases BACE1 expression	Murine microglial cells	(6)
**Cholesterol**	Induces β-secretase activity	Murine neuroblastoma cells	(15)
**Sphingolipids Sphingomyelin**	Induces γ-secretase activity	Purified human γ-secretase out of CHO cell	(16,17)
**Phosphatidylinositol 4,5-diphosphate**	Reduces γ-secretase activity	CHO cells	(18)
**Ceramide**	Induces β-secretase activity	CHO cells	(19)
**Phosphatidylinositol 3-kinase**	Sorting to intraluminal vesicles instead of endosomes resulting in the degradation of APP by the lysosomes	Murine neurons and human HeLa cells	(20)
**Phospholipase D1**	Disrupts γ-secretase target association	Murine neuroblastoma cells	(21)
**Hypoperfusion**	Increases γ-/β-secretase activity and γ-secretase expression	Murine brain	(22)
**Ischemia-reperfusion**	Increases γ-secretase activity	Murine brain tissues and human breast cancer cells	(23,24)
**Ischemia**	Increased β-secretase activity	Rat brains tissues	(25)
**Hypoxia**	Increases BACE1 expression	Murine neuroblastoma cell	(26)
**Phosphorylation by p25/Cdk5**	Increases BACE1 activity	Rat pheochromocytoma cells and murine brain tissue	(27)
**Acetylation by ATase1, ATase2**	Reduces BACE1 expression and activity	Human neuroblastoma cells	(28)
**HIV-1 Gag**	Induces γ-secretase activity	Human embryonic kidney and monocyte cell lines	(29)
**Chlamydia pneumoniae**	Increases BACE1 and PSEN1 expression	Human astrocytes	(30)
Abbreviations: ApoE: Apolipoprotein E; APP: amyloid precursor protein; Aβ: amyloid beta; ARF6: ADP ribosylation factor 6; BACE1: Beta secretase 1; Cdk5: cyclin-dependent kinase 5; CHO cells: Chinese hamster ovary cells; HIV: human immunodeficiency virus; IFN-γ: Interferon gamma; IL-1β: Interleukin-1 beta; IL-6: Interleukin-6; IL-6R:			

Interleukin-6 receptor; LPS: Lipopolysaccharide; PSEN1: Presenilin 1; SIRT1: Sirtuin 1; TNF-α: Tumor Necrosis Factor alpha.

**Supplemental Table 2. Determinants of amyloidogenic Aβ metabolism**			
**Aβ clearance**			
**Effector**	**Effect**	**Tissue / cell specificity**	**Source**
**Aging**	↓	Rat brains tissues	(31-33)
**Sleep**	↑	Murine brain tissue	(34)
**ApoE4>ApoE3>ApoE2**	Increases perivascular Aβ1-40 and Aβ1-42 transport	Bioengineered bipartite vessels, murine blood brain barrier	(35,36)
**HDL**	Increases perivascular Aβ1-40 and Aβ1-42 transport	Bioengineered bipartite vessels	(35)
**ABCA1**	↑	Murine brain tissue	(37-39)
**LRP1**	↓	Brains of Dutch/Iowa APP and APPsw± mice studies	(40)
**MYOCD**	↓	Pial arteries of Dutch/Iowa APP and APPsw± mice studies	(40)
**SORL1**	Reduces soluble Aβ1-40 and Aβ1-42 expression	SORL1 cDNA overexpressing HiPSC-derived neurons	(41)
**RAGE**	Influx of Aβ1-40 and Aβ1-42 from brain capillary across BBB	Murine brains	(42,43)
**Scara1**	Increased sAβ uptake	Murine brains, monocytes and microglia	(44)
**TREM2**	↑	Murine microglial ells	(45)
**LXR**	↑	Murine neuroblastoma cells	(46)
**Protein Kinase C**	TFEB-mediated Lysosomal clearance of Aβ	Murine brain tissue	(47,48)
**Dynamin-mediated endocytosis**	↑	Murine brain, fibroblast cells, hamster ovarian cells, human neuroblastoma and embryonic kidney cells	(49-51)
**LC3-associated endocytosis (LANDO)**	↑	Murine microglial ells	(52)
**Renal failure**	↓	Human blood plasma samples	(53)
**Hemodialysis**	↑	Human blood plasma samples	(54)
**Aβ degradation**			
**MMP2**	Degradation of Aβ1-40 and Aβ1-42	Recombinant human molecules MMP2	(55)
**MMP9**	Degradation of Aβ1-40 and Aβ1-42	Recombinant human molecules MMP9	(55)
**MMP14**	Degradation of Aβ1-40 and Aβ1-42	Monkey COS-1 cells	(56)
**Cathepsin D**	Degradation of Aβ1-40	Human non-neurological ill brains and recombinant molecules	(57)
**ACE**	Degradation of Aβ1-40 and Aβ1-42	Recombinant human molecules and murine brains and cortical vessels	(58-60)
**Neprilysin**	Degradation of Aβ1-40 and Aβ1-42	Mice brain tissues	(61)
**Insulin degrading enzyme**	Degradation of Aβ	Murine microglial cells	(62,63)

Abbreviations: ABCA1: ATP-binding cassette transporter; ApoE: Apolipoprotein E; APP: amyloid precursor protein; APPsw±: APP with the Swedish double mutation; ARF6: ADP-ribosylation factor 6; BBB: blood brain barrier ACE: Angiotensin Converting Enzyme; HiPSC: Human Induced Pluripotent Stem Cells; LRP1: Low Density Lipoprotein Receptor-related Protein 1; LXR: Liver X receptor; MMP: Matrix-Metalloprotease; MYOCD: myocardin; RAGE: receptor for advanced glycation end products; SORL1: Sortilin Related Receptor 1; TFEB: Transcription Factor EB HDL: High Density Lipoprotein; TREM2: Triggering receptor expressed on myeloid cells 2.

Supplemental Table 3. Diseases and other molecular and genetic determinants of Aβ abundance

Diseases associated with Aβ abundance and other molecular determinants

Effector	Effect	Tissue / cell specificity	Source
**Down Syndrome**	↑	Human hippocampal, prefrontal and frontal brain sections	(64,65)
**Cardiovascular disease**	Association with increased Aβ1-40	Human blood plasma probes	(66-68)
**Creutzfeldt–Jakob disease**	↑	Human brains	(69)
**anti-HSV IgM**	Association with reduced Aβ1-40 and Aβ1-42	Human blood plasma probes	(70)
**Liver cirrhosis**	Association with increased Aβ1-40 and Aβ1-42	Human blood plasma probes	(71)
**HBV**	Association with increased Aβ1-40 and Aβ1-42	Human blood plasma probes	(71)
**Obstructive sleep apnea syndrome**	Association with increased Aβ1-40 and Aβ1-42	Human blood plasma probes	(72)
**Chronic obstructive pulmonary disease**	Association with increased Aβ1-40 and Aβ1-42	Human blood plasma probes	(73)
**Infectious burden**	Association with increased Aβ1-40 and Aβ1-42	Human blood plasma probes	(74)
**Phosphorylation of Presenilin-1**	↓	Mouse with mutated phosphorylation sides PS1-S367A and PS1-S367D$^{-/-, -/+}$ and wildtype	(75)
**Mutant Presenilin-2 APP^{GFP} lysine-to-arginine**	↑	Murine neuroblastoma cells	(76)
**NLRP3**	↑	Murine brains	(77)
**IL-4/IL-13**	↓	Murine brains	(78)
**IL-12**	↑	Murine brains	(79)
**IL-17**	↓	Murine cerebrospinal fluids and hippocampus	(39)

Genetic determinants of Aβ abundance

APP mutation (SNP)	Tissue / cell specificity	Source
**A673V, K670N/M671L**	Increased Aβ generation studied in human embryonic kidney cells	(80,81)
**Lys670Asn and Met671Leu**	Increased Aβ generation studied in a human fibroblast cell line	(82)
**V717I**	Increased Aβ1-42 generation studied in mouse neuronal cells	(83)
**E682K**	Increased Aβ generation studied in murine primary cortical neurons and CHO cells	(84)
**A673T**	Reduced Aβ1-40 levels studied in human embryonic kidney cell	(80,81)
**E693G**	Reduced Aβ plasma concentration, but quicker Aβ protofibril formation studied in human blood plasma samples	(85)
**T714I, V715A, V715M, V717I or V717L**	Reduced Aβ1-40, but increased Aβ1-42 generation studied in murine primary cortical neurons	(84)
**A673V, K670N/M671L**	Increased Aβ generation studied in human embryonic kidney cells	(86)

Abbreviations: APP: amyloid precursor protein; Aβ: amyloid beta; HBV: Hepatitis B Virus; HSV: Herpes-simplex-Virus; IL-12: Interleukin-12; IL-13: Interleukin-13; IL-17: Interleukin-17; IL-4: Interleukin-4; SNP: Single Nucleotide Polymorphism.

Supplemental Table 4. Off target effects of lifestyle modifications on Aβ metabolism and accumulation.			
**Intervention/condition**	**Cell type/Population**	**Effects on Aβ metabolism**	**Source**
**Smoking**	APPswe/PS1dE9 transgenic mice	Smoking promotes the formation of new total Aβ deposits in neurons and microglial cells	(87)
**High fat diet**	APP transgenic mice	High fat diet promotes APP cleavage by BACE1 in neurons	(88)
**Mediterranean diet**	Prospective clinical study of 70 cognitively normal individuals 30- to 60-year-old	Mediterranean diet and adherence to it, are associated with lower total Aβ deposition in human brain	(89)
**Culture media rich in omega-3 fatty acids**	Human CHME3 microglial cells	Omega-3 fatty acids enhance phagocytosis of Aβ1-42 in human microglial cells	(90)
**Caloric restriction**	Tg2576 mice	Caloric restriction reduces γ-secretase expression, Aβ1-40(-79.7%) and Aβ1-42(-64.2%) peptide levels in neurons	(91)
**Physical activity**	Cross sectional study of 546 cognitively healthy adults	High physical activity is associated with lower plasma Aβ1-42/1-40 ratio, but not Aβ1-40 and Aβ1-42	(92)
**Physical activity**	Prospective clinical study of 149 cognitively healthy older individuals.	Higher physical activity is associated with lower plasma Aβ1-42 and lower Aβ1-42/1-40 ratio, Aβ1-40 levels are not affected	(93)
**Daily activity VO2max**	Cross-sectional study of 3266 participants without CVD	Higher daily activity and VO2max associated with lower plasma Aβ1-40	(66)
Abbreviations: APP: amyloid precursor protein; Aβ: amyloid beta; BACE1: Beta-secretase 1; CVD: cardiovascular disease;			

Supplemental Table 5. Off target effects of anti-thrombotic agents on Aβ metabolism and accumulation.			
**Anticoagulants**			
**Intervention/condition**	**Cell type/Population**	**Effects on Aβ metabolism**	**Source**
**Heparin**	PC12 mice cells	Heparin attenuates toxic effects induced by Aβ	(94)
**Enoxaparin**	APP23 transgenic mice	Enoxaparin reduces Aβ and amyloid plaques in brain	(95)
**Heparin**	SHSY5Y neuroblastoma cells	Heparin inhibits BACE1 and APP cleavage in neurons	(96)
**Heparin**	Recombinant human BACE1 incubated with heparin	In low concentration, heparin stimulates BACE1 activity and its autocatalysis, while in high concentration, heparin inhibits BACE1	(97)
**Heparin**	Human neuroblastoma cells	Increased neuronal secretion of total APP and sAPPβ	(98)
**Heparin**	Recombinant human BACE1	Heparin activates proBACE1 greater than other GAGs. The amount of stimulation of activity was dependent upon the overall structure and charge of the GAGs.	(99)
**Rivaroxaban, Dabigatran, Warfarin, Enoxaparin**	C57BL/6 mice	Aβ1-40 and Aβ1-42 plasma levels are increased (>20fold) after treatment with rivaroxaban, dabigatran, warfarin, enoxaparin.	(100)
**Heparin**	Cortical cell culture of Tg2576 mice	Reduced neuronal Aβ1-40 secretion (maximum -30%)	(101)
**Heparin, Enoxaparin**	Cortical cell culture of Tg2576 mice	Decreased APP processing via non-specific inhibition of alpha and beta secretase pathways in neurons	(102)
**Antiplatelets**			
**Clopidogrel**	APP 23 mice, Human platelets	Reduced Aβ deposition in cerebral vascular wall (neurons and platelets)	(103)
**Aspirin**	Synthetic human Aβ peptides	Aspirin inhibits Aβ aggregation in vitro	(104)
**Aspirin**	Isolated human platelets	Inhibition of cyclooxygenase by aspirin on human platelets reduces APP but not Aβ release	(105)
**Low-dose aspirin**	5X familial Alzheimer's disease transgenic mice	Low-dose aspirin decreases amyloid plaques through a PPARα-dependent mechanism in neurons	(106)
Abbreviations: Aβ: amyloid beta; APP: amyloid precursor protein; BACE1: Beta-secretase 1; sAPPβ: soluble peptide APPβ; GAGs: glycosaminoglycans; PPARα: Peroxisome proliferator-activated receptor α			

Table References

1. Nordstedt C, Gandy SE, Alafuzoff I et al. Alzheimer beta/A4 amyloid precursor protein in human brain: aging-associated increases in holoprotein and in a proteolytic fragment. Proc Natl Acad Sci U S A 1991;88:8910-4.
2. Goldgaber D, Harris HW, Hla T et al. Interleukin 1 regulates synthesis of amyloid beta-protein precursor mRNA in human endothelial cells. Proc Natl Acad Sci U S A 1989;86:7606-10.
3. Forloni G, Demicheli F, Giorgi S, Bendotti C, Angeretti N. Expression of amyloid precursor protein mRNAs in endothelial, neuronal and glial cells: modulation by interleukin-1. Brain Res Mol Brain Res 1992;16:128-34.
4. Ringheim GE, Szczepanik AM, Petko W, Burgher KL, Zhu SZ, Chao CC. Enhancement of beta-amyloid precursor protein transcription and expression by the soluble interleukin-6 receptor/interleukin-6 complex. Brain Res Mol Brain Res 1998;55:35-44.
5. Chung J, Phukan G, Vergote D et al. Endosomal-Lysosomal Cholesterol Sequestration by U18666A Differentially Regulates Amyloid Precursor Protein (APP) Metabolism in Normal and APP-Overexpressing Cells. Mol Cell Biol 2018;38.
6. Badshah H, Ali T, Kim MO. Osmotin attenuates LPS-induced neuroinflammation and memory impairments via the TLR4/NFkappaB signaling pathway. Sci Rep 2016;6:24493.
7. Sawamura N, Ko M, Yu W et al. Modulation of amyloid precursor protein cleavage by cellular sphingolipids. J Biol Chem 2004;279:11984-91.
8. Huang YA, Zhou B, Wernig M, Sudhof TC. ApoE2, ApoE3, and ApoE4 Differentially Stimulate APP Transcription and Abeta Secretion. Cell 2017;168:427-441 e21.
9. Shi J, Yang SH, Stubley L, Day AL, Simpkins JW. Hypoperfusion induces overexpression of beta-amyloid precursor protein mRNA in a focal ischemic rodent model. Brain Res 2000;853:1-4.
10. Fukumoto H, Rosene DL, Moss MB, Raju S, Hyman BT, Irizarry MC. Beta-secretase activity increases with aging in human, monkey, and mouse brain. Am J Pathol 2004;164:719-25.
11. Hong HS, Hwang EM, Sim HJ et al. Interferon gamma stimulates beta-secretase expression and sAPPbeta production in astrocytes. Biochem Biophys Res Commun 2003;307:922-7.
12. Yamamoto M, Kiyota T, Horiba M et al. Interferon-gamma and tumor necrosis factor-alpha regulate amyloid-beta plaque deposition and beta-secretase expression in Swedish mutant APP transgenic mice. Am J Pathol 2007;170:680-92.
13. Sannerud R, Declerck I, Peric A et al. ADP ribosylation factor 6 (ARF6) controls amyloid precursor protein (APP) processing by mediating the endosomal sorting of BACE1. Proc Natl Acad Sci U S A 2011;108:E559-68.
14. Qin W, Yang T, Ho L et al. Neuronal SIRT1 activation as a novel mechanism underlying the prevention of Alzheimer disease amyloid neuropathology by calorie restriction. J Biol Chem 2006;281:21745-54.
15. Ehehalt R, Keller P, Haass C, Thiele C, Simons K. Amyloidogenic processing of the Alzheimer beta-amyloid precursor protein depends on lipid rafts. J Cell Biol 2003;160:113-23.
16. Osenkowski P, Ye W, Wang R, Wolfe MS, Selkoe DJ. Direct and potent regulation of gamma-secretase by its lipid microenvironment. J Biol Chem 2008;283:22529-40.
17. Grimm MO, Grimm HS, Patzold AJ et al. Regulation of cholesterol and sphingomyelin metabolism by amyloid-beta and presenilin. Nat Cell Biol 2005;7:1118-23.
18. Osawa S, Funamoto S, Nobuhara M et al. Phosphoinositides suppress gamma-secretase in both the detergent-soluble and -insoluble states. J Biol Chem 2008;283:19283-92.

19. Puglielli L, Ellis BC, Saunders AJ, Kovacs DM. Ceramide stabilizes beta-site amyloid precursor protein-cleaving enzyme 1 and promotes amyloid beta-peptide biogenesis. J Biol Chem 2003;278:19777-83.

20. Morel E, Chamoun Z, Lasiecka ZM et al. Phosphatidylinositol-3-phosphate regulates sorting and processing of amyloid precursor protein through the endosomal system. Nat Commun 2013;4:2250.

21. Cai D, Netzer WJ, Zhong M et al. Presenilin-1 uses phospholipase D1 as a negative regulator of beta-amyloid formation. Proc Natl Acad Sci U S A 2006;103:1941-6.

22. Wang L, Du Y, Wang K, Xu G, Luo S, He G. Chronic cerebral hypoperfusion induces memory deficits and facilitates Abeta generation in C57BL/6J mice. Exp Neurol 2016;283:353-64.

23. Arumugam TV, Chan SL, Jo DG et al. Gamma secretase-mediated Notch signaling worsens brain damage and functional outcome in ischemic stroke. Nat Med 2006;12:621-3.

24. Villa JC, Chiu D, Brandes AH et al. Nontranscriptional role of Hif-1alpha in activation of gamma-secretase and notch signaling in breast cancer. Cell Rep 2014;8:1077-92.

25. Wen Y, Onyewuchi O, Yang S, Liu R, Simpkins JW. Increased beta-secretase activity and expression in rats following transient cerebral ischemia. Brain Res 2004;1009:1-8.

26. Zhang X, Zhou K, Wang R et al. Hypoxia-inducible factor 1alpha (HIF-1alpha)-mediated hypoxia increases BACE1 expression and beta-amyloid generation. J Biol Chem 2007;282:10873-80.

27. Song WJ, Son MY, Lee HW, Seo H, Kim JH, Chung SH. Enhancement of BACE1 Activity by p25/Cdk5-Mediated Phosphorylation in Alzheimer's Disease. PLoS One 2015;10:e0136950.

28. Ding Y, Ko MH, Pehar M et al. Biochemical inhibition of the acetyltransferases ATase1 and ATase2 reduces beta-secretase (BACE1) levels and Abeta generation. J Biol Chem 2012;287:8424-33.

29. Chai Q, Jovasevic V, Malikov V et al. HIV-1 counteracts an innate restriction by amyloid precursor protein resulting in neurodegeneration. Nat Commun 2017;8:1522.

30. Al-Atrache Z, Lopez DB, Hingley ST, Appelt DM. Astrocytes infected with Chlamydia pneumoniae demonstrate altered expression and activity of secretases involved in the generation of beta-amyloid found in Alzheimer disease. BMC Neurosci 2019;20:6.

31. Dewachter I, Van Dorpe J, Smeijers L et al. Aging increased amyloid peptide and caused amyloid plaques in brain of old APP/V717I transgenic mice by a different mechanism than mutant presenilin1. J Neurosci 2000;20:6452-8.

32. Silverberg GD, Miller MC, Messier AA et al. Amyloid deposition and influx transporter expression at the blood-brain barrier increase in normal aging. J Neuropathol Exp Neurol 2010;69:98-108.

33. Silverberg GD, Messier AA, Miller MC et al. Amyloid efflux transporter expression at the blood-brain barrier declines in normal aging. J Neuropathol Exp Neurol 2010;69:1034-43.

34. Kang JE, Lim MM, Bateman RJ et al. Amyloid-beta dynamics are regulated by orexin and the sleep-wake cycle. Science 2009;326:1005-7.

35. Robert J, Button EB, Yuen B et al. Clearance of beta-amyloid is facilitated by apolipoprotein E and circulating high-density lipoproteins in bioengineered human vessels. Elife 2017;6.

36. Deane R, Sagare A, Hamm K et al. apoE isoform-specific disruption of amyloid beta peptide clearance from mouse brain. J Clin Invest 2008;118:4002-13.

37. Koldamova R, Staufenbiel M, Lefterov I. Lack of ABCA1 considerably decreases brain ApoE level and increases amyloid deposition in APP23 mice. J Biol Chem 2005;280:43224-35.

38. Wahrle SE, Jiang H, Parsadanian M et al. Deletion of Abca1 increases Abeta deposition in the PDAPP transgenic mouse model of Alzheimer disease. J Biol Chem 2005;280:43236-42.

39. Yang J, Kou J, Lalonde R, Fukuchi KI. Intracranial IL-17A overexpression decreases cerebral amyloid angiopathy by upregulation of ABCA1 in an animal model of Alzheimer's disease. Brain Behav Immun 2017;65:262-273.

40. Bell RD, Deane R, Chow N et al. SRF and myocardin regulate LRP-mediated amyloid-beta clearance in brain vascular cells. Nat Cell Biol 2009;11:143-53.

41. Young JE, Boulanger-Weill J, Williams DA et al. Elucidating molecular phenotypes caused by the SORL1 Alzheimer's disease genetic risk factor using human induced pluripotent stem cells. Cell Stem Cell 2015;16:373-85.

42. Yan SD, Chen X, Fu J et al. RAGE and amyloid-beta peptide neurotoxicity in Alzheimer's disease. Nature 1996;382:685-91.

43. Deane R, Du Yan S, Submamaryan RK et al. RAGE mediates amyloid-beta peptide transport across the blood-brain barrier and accumulation in brain. Nat Med 2003;9:907-13.

44. Frenkel D, Wilkinson K, Zhao L et al. Scara1 deficiency impairs clearance of soluble amyloid-beta by mononuclear phagocytes and accelerates Alzheimer's-like disease progression. Nat Commun 2013;4:2030.

45. Zhao Y, Wu X, Li X et al. TREM2 Is a Receptor for beta-Amyloid that Mediates Microglial Function. Neuron 2018;97:1023-1031 e7.

46. Sun Y, Yao J, Kim TW, Tall AR. Expression of liver X receptor target genes decreases cellular amyloid beta peptide secretion. J Biol Chem 2003;278:27688-94.

47. Li Y, Xu M, Ding X et al. Protein kinase C controls lysosome biogenesis independently of mTORC1. Nat Cell Biol 2016;18:1065-77.

48. Xiao Q, Yan P, Ma X et al. Neuronal-Targeted TFEB Accelerates Lysosomal Degradation of APP, Reducing Abeta Generation and Amyloid Plaque Pathogenesis. J Neurosci 2015;35:12137-51.

49. Wesen E, Jeffries GDM, Matson Dzebo M, Esbjorner EK. Endocytic uptake of monomeric amyloid-beta peptides is clathrin- and dynamin-independent and results in selective accumulation of Abeta(1-42) compared to Abeta(1-40). Sci Rep 2017;7:2021.

50. Cirrito JR, Kang JE, Lee J et al. Endocytosis is required for synaptic activity-dependent release of amyloid-beta in vivo. Neuron 2008;58:42-51.

51. Carey RM, Balcz BA, Lopez-Coviella I, Slack BE. Inhibition of dynamin-dependent endocytosis increases shedding of the amyloid precursor protein ectodomain and reduces generation of amyloid beta protein. BMC Cell Biol 2005;6:30.

52. Heckmann BL, Teubner BJW, Tummers B et al. LC3-Associated Endocytosis Facilitates beta-Amyloid Clearance and Mitigates Neurodegeneration in Murine Alzheimer's Disease. Cell 2019;178:536-551 e14.

53. Arvanitakis Z, Lucas JA, Younkin LH, Younkin SG, Graff-Radford NR. Serum creatinine levels correlate with plasma amyloid Beta protein. Alzheimer Dis Assoc Disord 2002;16:187-90.

54. Rubio I, Caramelo C, Gil A, Lopez MD, de Yebenes JG. Plasma amyloid-beta, Abeta1-42, load is reduced by haemodialysis. J Alzheimers Dis 2006;10:439-43.

55. Hernandez-Guillamon M, Mawhirt S, Blais S et al. Sequential Amyloid-beta Degradation by the Matrix Metalloproteases MMP-2 and MMP-9. J Biol Chem 2015;290:15078-91.

56. Liao MC, Van Nostrand WE. Degradation of soluble and fibrillar amyloid beta-protein by matrix metalloproteinase (MT1-MMP) in vitro. Biochemistry 2010;49:1127-36.

57. McDermott JR, Gibson AM. Degradation of Alzheimer's beta-amyloid protein by human cathepsin D. Neuroreport 1996;7:2163-6.

58. Hu J, Igarashi A, Kamata M, Nakagawa H. Angiotensin-converting enzyme degrades Alzheimer amyloid beta-peptide (A beta); retards A beta aggregation, deposition, fibril formation; and inhibits cytotoxicity. J Biol Chem 2001;276:47863-8.

59. Eckman EA, Watson M, Marlow L, Sambamurti K, Eckman CB. Alzheimer's disease beta-amyloid peptide is increased in mice deficient in endothelin-converting enzyme. J Biol Chem 2003;278:2081-4.

60. Bernstein KE, Koronyo Y, Salumbides BC et al. Angiotensin-converting enzyme overexpression in myelomonocytes prevents Alzheimer's-like cognitive decline. J Clin Invest 2014;124:1000-12.

61. Iwata N, Tsubuki S, Takaki Y et al. Metabolic regulation of brain Abeta by neprilysin. Science 2001;292:1550-2.

62. Qiu WQ, Walsh DM, Ye Z et al. Insulin-degrading enzyme regulates extracellular levels of amyloid beta-protein by degradation. J Biol Chem 1998;273:32730-8.

63. Kurochkin IV, Goto S. Alzheimer's beta-amyloid peptide specifically interacts with and is degraded by insulin degrading enzyme. FEBS Lett 1994;345:33-7.

64. Wisniewski KE, Dalton AJ, McLachlan C, Wen GY, Wisniewski HM. Alzheimer's disease in Down's syndrome: clinicopathologic studies. Neurology 1985;35:957-61.

65. Teller JK, Russo C, DeBusk LM et al. Presence of soluble amyloid beta-peptide precedes amyloid plaque formation in Down's syndrome. Nat Med 1996;2:93-5.

66. Stamatelopoulos K, Pol CJ, Ayers C et al. Amyloid-Beta (1-40) Peptide and Subclinical Cardiovascular Disease. J Am Coll Cardiol 2018;72:1060-1.

67. Stamatelopoulos K, Mueller-Hennessen M, Georgiopoulos G et al. Amyloid-beta (1-40) and Mortality in Patients With Non-ST-Segment Elevation Acute Coronary Syndrome: A Cohort Study. Ann Intern Med 2018;168:855-65.

68. Stamatelopoulos K, Sibbing D, Rallidis LS et al. Amyloid-beta (1-40) and the risk of death from cardiovascular causes in patients with coronary heart disease. J Am Coll Cardiol 2015;65:904-16.

69. Jaunmuktane Z, Mead S, Ellis M et al. Evidence for human transmission of amyloid-beta pathology and cerebral amyloid angiopathy. Nature 2015;525:247-50.

70. Feart C, Helmer C, Fleury H et al. Association between IgM anti-herpes simplex virus and plasma amyloid-beta levels. PLoS One 2011;6:e29480.

71. Wang YR, Wang QH, Zhang T et al. Associations Between Hepatic Functions and Plasma Amyloid-Beta Levels-Implications for the Capacity of Liver in Peripheral Amyloid-Beta Clearance. Mol Neurobiol 2017;54:2338-2344.

72. Bu XL, Liu YH, Wang QH et al. Serum amyloid-beta levels are increased in patients with obstructive sleep apnea syndrome. Sci Rep 2015;5:13917.

73. Bu XL, Cao GQ, Shen LL et al. Serum Amyloid-Beta Levels are Increased in Patients with Chronic Obstructive Pulmonary Disease. Neurotox Res 2015;28:346-51.

74. Bu XL, Yao XQ, Jiao SS et al. A study on the association between infectious burden and Alzheimer's disease. Eur J Neurol 2015;22:1519-25.

75. Bustos V, Pulina MV, Kelahmetoglu Y et al. Bidirectional regulation of Abeta levels by Presenilin 1. Proc Natl Acad Sci U S A 2017;114:7142-7147.

76. Williamson RL, Laulagnier K, Miranda AM et al. Disruption of amyloid precursor protein ubiquitination selectively increases amyloid beta (Abeta) 40 levels via presenilin 2-mediated cleavage. J Biol Chem 2017;292:19873-19889.

77. Heneka MT, Kummer MP, Stutz A et al. NLRP3 is activated in Alzheimer's disease and contributes to pathology in APP/PS1 mice. Nature 2013;493:674-8.

78. Kawahara K, Suenobu M, Yoshida A et al. Intracerebral microinjection of interleukin-4/interleukin-13 reduces beta-amyloid accumulation in the ipsilateral side and improves cognitive deficits in young amyloid precursor protein 23 mice. Neuroscience 2012;207:243-60.

79. Vom Berg J, Prokop S, Miller KR et al. Inhibition of IL-12/IL-23 signaling reduces Alzheimer's disease-like pathology and cognitive decline. Nat Med 2012;18:1812-9.

80. Jonsson T, Atwal JK, Steinberg S et al. A mutation in APP protects against Alzheimer's disease and age-related cognitive decline. Nature 2012;488:96-9.

81. Maloney JA, Bainbridge T, Gustafson A et al. Molecular mechanisms of Alzheimer disease protection by the A673T allele of amyloid precursor protein. J Biol Chem 2014;289:30990-1000.

82. Citron M, Vigo-Pelfrey C, Teplow DB et al. Excessive production of amyloid beta-protein by peripheral cells of symptomatic and presymptomatic patients carrying the Swedish familial Alzheimer disease mutation. Proc Natl Acad Sci U S A 1994;91:11993-7.

83. Muratore CR, Rice HC, Srikanth P et al. The familial Alzheimer's disease APPV717I mutation alters APP processing and Tau expression in iPSC-derived neurons. Hum Mol Genet 2014;23:3523-36.

84. Zhou L, Brouwers N, Benilova I et al. Amyloid precursor protein mutation E682K at the alternative beta-secretase cleavage beta'-site increases Abeta generation. EMBO Mol Med 2011;3:291-302.

85. Nilsberth C, Westlind-Danielsson A, Eckman CB et al. The 'Arctic' APP mutation (E693G) causes Alzheimer's disease by enhanced Abeta protofibril formation. Nat Neurosci 2001;4:887-93.

86. De Jonghe C, Esselens C, Kumar-Singh S et al. Pathogenic APP mutations near the gamma-secretase cleavage site differentially affect Abeta secretion and APP C-terminal fragment stability. Hum Mol Genet 2001;10:1665-71.

87. Moreno-Gonzalez I, Estrada LD, Sanchez-Mejias E, Soto C. Smoking exacerbates amyloid pathology in a mouse model of Alzheimer's disease. Nat Commun 2013;4:1495.

88. Maesako M, Uemura M, Tashiro Y et al. High Fat Diet Enhances beta-Site Cleavage of Amyloid Precursor Protein (APP) via Promoting beta-Site APP Cleaving Enzyme 1/Adaptor Protein 2/Clathrin Complex Formation. PLoS One 2015;10:e0131199.

89. Berti V, Walters M, Sterling J et al. Mediterranean diet and 3-year Alzheimer brain biomarker changes in middle-aged adults. Neurology 2018;90:e1789-e1798.

90. Hjorth E, Zhu M, Toro VC et al. Omega-3 fatty acids enhance phagocytosis of Alzheimer's disease-related amyloid-beta42 by human microglia and decrease inflammatory markers. J Alzheimers Dis 2013;35:697-713.

91. Schafer MJ, Alldred MJ, Lee SH et al. Reduction of beta-amyloid and gamma-secretase by calorie restriction in female Tg2576 mice. Neurobiol Aging 2015;36:1293-302.

92. Brown BM, Peiffer JJ, Taddei K et al. Physical activity and amyloid-beta plasma and brain levels: results from the Australian Imaging, Biomarkers and Lifestyle Study of Ageing. Mol Psychiatry 2013;18:875-81.

93. Stillman CM, Lopez OL, Becker JT et al. Physical activity predicts reduced plasma beta amyloid in the Cardiovascular Health Study. Ann Clin Transl Neurol 2017;4:284-291.

94. Pollack SJ, Sadler, II, Hawtin SR, Tailor VJ, Shearman MS. Sulfated glycosaminoglycans and dyes attenuate the neurotoxic effects of beta-amyloid in rat PC12 cells. Neurosci Lett 1995;184:113-6.

95. Bergamaschini L, Rossi E, Storini C et al. Peripheral treatment with enoxaparin, a low molecular weight heparin, reduces plaques and beta-amyloid accumulation in a mouse model of Alzheimer's disease. J Neurosci 2004;24:4181-6.

96. Scholefield Z, Yates EA, Wayne G, Amour A, McDowell W, Turnbull JE. Heparan sulfate regulates amyloid precursor protein processing by BACE1, the Alzheimer's beta-secretase. J Cell Biol 2003;163:97-107.

97. Beckman M, Holsinger RM, Small DH. Heparin activates beta-secretase (BACE1) of Alzheimer's disease and increases autocatalysis of the enzyme. Biochemistry 2006;45:6703-14.

98. Leveugle B, Ding W, Durkin JT et al. Heparin promotes beta-secretase cleavage of the Alzheimer's amyloid precursor protein. Neurochem Int 1997;30:543-8.

99. Klaver DW, Wilce MC, Gasperini R et al. Glycosaminoglycan-induced activation of the beta-secretase (BACE1) of Alzheimer's disease. J Neurochem 2010;112:1552-61.

100. Yang L, Bhattacharya A, Li Y, Zhang Y. Anticoagulants inhibit proteolytic clearance of plasma amyloid beta. Oncotarget 2018;9:5614-26.

101. Klaver D, Hung AC, Gasperini R, Foa L, Aguilar MI, Small DH. Effect of heparin on APP metabolism and Abeta production in cortical neurons. Neurodegener Dis 2010;7:187-9.

102. Cui H, Hung AC, Klaver DW et al. Effects of heparin and enoxaparin on APP processing and Abeta production in primary cortical neurons from Tg2576 mice. PLoS One 2011;6:e23007.

103. Donner L, Falker K, Gremer L et al. Platelets contribute to amyloid-beta aggregation in cerebral vessels through integrin alphaIIbbeta3-induced outside-in signaling and clusterin release. Sci Signal 2016;9:ra52.

104. Thomas T, Nadackal TG, Thomas K. Aspirin and non-steroidal anti-inflammatory drugs inhibit amyloid-beta aggregation. Neuroreport 2001;12:3263-7.

105. Skovronsky DM, Lee VM, Pratico D. Amyloid precursor protein and amyloid beta peptide in human platelets. Role of cyclooxygenase and protein kinase C. J Biol Chem 2001;276:17036-43.

106. Chandra S, Jana M, Pahan K. Aspirin Induces Lysosomal Biogenesis and Attenuates Amyloid Plaque Pathology in a Mouse Model of Alzheimer's Disease via PPARalpha. J Neurosci 2018;38:6682-6699.
